# Hemolytic and Antimicrobial Activities of a Series of Cationic Amphiphilic Copolymers Comprised of Same Centered Comonomers with Thiazole Moieties and Polyethylene Glycol Derivatives

**DOI:** 10.3390/polym12040972

**Published:** 2020-04-22

**Authors:** R. Cuervo-Rodríguez, A. Muñoz-Bonilla, F. López-Fabal, M. Fernández-García

**Affiliations:** 1 Facultad de Ciencias Químicas, Universidad Complutense de Madrid, Avenida Complutense s/n, Ciudad Universitaria, 28040 Madrid, Spain; rociocr@quim.ucm.es; 2 Instituto de Ciencia y Tecnología de Polímeros (ICTP-CSIC), C/Juan de la Cierva 3, 28006 Madrid, Spain; martafg@ictp.csic.es; 3 Interdisciplinary Platform for Sustainable Plastics towards a Circular Economy-Spanish National Research Council (SusPlast-CSIC), 28006 Madrid, Spain; 4 Hospital Universitario de Móstoles C/ Luis Montes, s/n, 28935 Madrid, Spain; flopezf@salud.madrid.org

**Keywords:** antimicrobial polymers, amphiphilic polymers, hemolytic activity, hemocompatibility, PEGMA, thiazole

## Abstract

A series of well-defined antimicrobial polymers composed of comonomers bearing thiazole ring (2-(((2-(4-methylthiazol-5-yl)ethoxy)carbonyl)oxy)ethyl methacrylate monomer (MTZ)) and non-hemotoxic poly(ethylene glycol) side chains (poly(ethylene glycol) methyl ether methacrylate (PEGMA)) were synthesized by reversible addition-fragmentation chain transfer (RAFT) polymerization. By post-polymerization functionalization strategy, polymers were quaternized with either butyl or octyl iodides to result in cationic amphiphilic copolymers incorporating thiazolium groups, thus with variable hydrophobic/hydrophilic balance associated to the length of the alkylating agent. Likewise, the molar percentage of PEGMA was modulated in the copolymers, also affecting the amphiphilicity. The antimicrobial activities of these cationic polymers were determined against Gram-positive and Gram-negative bacteria and fungi. Minimum inhibitory concentration (MIC) was found to be dependent on both length of the alkyl hydrophobic chain and the content of PEGMA in the copolymers. More hydrophobic octylated copolymers were found to be more effective against all tested microorganisms. The incorporation of non-ionic hydrophilic units, PEGMA, reduces the hydrophobicity of the system and the activity is markedly reduced. This effect is dramatic in the case of butylated copolymers, in which the hydrophobic/hydrophilic balance is highly affected. The hemolytic properties of polymers analyzed against human red blood cells were greatly affected by the hydrophobic/hydrophilic balance of the copolymers and the content of PEGMA, which drastically reduces the hemotoxicity. The copolymers containing longer hydrophobic chain, octyl, are much more hemotoxic than their corresponding butylated copolymers.

## 1. Introduction

Amphiphilic cationic polymers mimicking natural host defense peptides (HDPs) are a very promising class of antimicrobial compounds with unique characteristics for fighting microbial infections, involving a membrane-disruptive mode of action, which makes it difficult for bacteria to develop resistance [1,2,3,4,5]. Initially, these amphiphilic cationic polymers interact with microbial membranes through electrostatic interactions between cationic charge of polymers and anionic charge on the membrane surface; then, the hydrophobic parts allow the insertion of the polymers into membrane lipid regions, disrupting membrane structure [6]. One of the key priorities in the development of new antimicrobial polymers is to achieve broad-spectrum activity. Bacterial and fungal cell membranes exhibit an overall negative charge; however, a very wide variation in the cellular structure and organization is found among different kinds of microbes [7]. Gram-positive bacteria possess a thick and porous layer of peptidoglycan that gives a negative charge surrounding the cytoplasmic membrane, whereas Gram-negative bacteria have an outer membrane, surrounding a thinner peptidoglycan layer [8]. This outer layer contains lipopolysaccharide chains with negative charge. Related to fungi, the permeability of the membrane is in between Gram-positive and Gram-negative bacteria, is composed of polysaccharides, and does not have a marked negative charge. Therefore, these differences in cell walls play a crucial role in the susceptibility of microbes to cationic polymers. Typically, Gram-negative and fungi are more difficult to eliminate than Gram-positive [9], but contrary behaviors have been proven for some cationic polymeric systems [10] and broad-spectrum antimicrobial polymers have also been developed [11,12,13]. Some studies concluded that cationic charge influences more the interaction with the bacterial membrane of Gram-positive bacteria in comparison with Gram-negative bacteria, while the hydrophobicity and amphiphilic balance of the polymers affects more the efficacy against Gram-negative bacteria [6,14]. However, this is not an established rule and, in addition to the hydrophobic/hydrophilic balance and positive charge density, molecular weight, nature of the charges, and polymer architectures are other parameters that determine the efficiency of the antimicrobial polymers.

Besides the antimicrobial activity, the toxicity towards mammalian cells is a key factor for the clinical applicability of the antimicrobial polymers. Contrary to the microbial membrane, the nature of mammalian cell membrane is zwitterionic, and thus the cationic polymers preferably interact with the microbial membrane over the mammalian membrane [4]. However, the toxicity toward host mammalian cells is not always as low as desired, in particular in polymers with high hydrophobic contents [15]. Attempts to improve the biocompatibility of antimicrobial polymers include the incorporation of non-ionic and biocompatible moieties such as polyethylene glycol (PEG) [16,17,18,19] and glycopolymers [20,21]. The toxicity of cationic amphiphilic polymers is typically evaluated by the hemolytic activity towards mammalian red blood cells (RBCs). This is a more susceptible situation in comparison with real blood, as the plasma proteins adsorbed to the membrane provide a protective layer that prevents the contact with other compounds. Then, isolated RBCs are more susceptible to the action of antimicrobial polymers. Similarly, compounds such as PEG may interact with the RBC membranes through hydrogen bonds, exerting a protecting effect [22]. Although PEG functionalized polymers demonstrated a reduced toxicity, in many cases, the antimicrobial activity concomitantly decreases as the content of PEG augments in the polymer [23], mainly because the incorporation of a third component affects the amphiphilic balance of the polymer.

Considering the above discussion, the design of antimicrobial polymers with negligible toxicity and antibacterial effectiveness against a broad spectrum of microorganisms requires understanding the effect of structural polymer parameters on hemolytic and antibacterial activities of amphiphilic polymers. Our present investigation involves the preparation of well-defined copolymers composed of cationic amphiphilic units (MTZ-RI) and nonionic, biocompatible, and water soluble poly(ethylene glycol) methyl ether methacrylate (PEGMA) units. The cationic amphiphilic monomeric units are considered “same centered” units, in which a long hydrophobic alkyl chain is directly attached to the positively charged nitrogen moiety. In this case, the alkyl chain length (butyl or octyl) is varied to adjust the hydrophobic/hydrophilic balance. The amphiphilicity is also modulated by the percentage of PEGMA in the copolymer.

## 2. Experimental Part

### 2.1. Materials

2-(((2-(4-Methylthiazol-5-yl)ethoxy)carbonyl)oxy)ethyl methacrylate monomer, MTZ, was synthesized according to the procedure described previously [12]. Poly(ethylene glycol) methyl ether methacrylate (PEGMA, average *M*_n_ = 300 g/mol; Sigma-Aldrich, St Quentin Fallavier, France), 2-cyano-2-propyl benzodithioate (CPB, Sigma-Aldrich, >97%), anhydrous toluene (Sigma-Aldrich, 99.8%), anhydrous *N,N*-dimethylformamide (DMF, Sigma-Aldrich 99.8%), 1-iodooctane (Sigma-Aldrich, 98%), 1-iodobutane (Sigma Aldrich, 99%), pentane (Scharlau, 98%, Barcelona, Spain), and hexane (Scharlau, 99%) were used as received. 2,2’-Azobisisobutyronitrile (AIBN, Acros, 98%, Somerville, NJ, USA) radical initiator was purified by recrystallization in methanol. Cellulose dialysis membranes (CelluSep T-series and H1) were obtained from Membrane Filtration Products, Inc (Seguin, TX, USA).

*Microbiological and hemolysis assays:* NaCl solution (0.9%, BioXtra, suitable for cell culture), phosphate buffered saline powder (PBS, pH 7.4), Triton™ X-100 solution (BioUltra, for molecular biology, ~10% in H_2_O), and dimethylsulfoxide (DMSO, BioReagent, for molecular biology, suitable for plant cell culture, ≥99.9%) were all obtained from Aldrich. Sterile distilled water (Versylene) was purchased from Fresenius Kabi (Barcelona, Spain). BBL^TM^ Mueller Hinton broth used as growth media was purchased from Becton, Dickinson, and Company. Antimicrobial tests to determine minimal inhibitory concentration (MIC) were performed in 96-well microplates (BD Biosciences). The following from the American Type Culture Collection (ATCC) were purchased from Oxoid^TM^: *Staphylococcus aureus* (*S. aureus*, ATCC^®^ 29213), *Staphylococcus epidermidis* (*S. epidermidis*, ATCC^®^ 12228), *Escherichia coli* (*E. coli*, ATCC^®^ 25922), *Pseudomonas aeruginosa* (*P. aeruginosa*, ATCC^®^ 27853) bacteria, and *Candida parapsilosis* (*C. parapsilosis*, ATCC^®^ 22109) yeast. The optical density of the microorganism suspensions to estimate the microorganism concentration was measured in McFarland units proportional by a DensiCHEK^TM^ Plus (VITEK^®^, BioMérieux). Human blood was taken from healthy donors (Hospital Universitario de Móstoles, Madrid, Spain).

### 2.2. Instrumentations

^1^H and ^13^C nuclear magnetic resonance (NMR) spectra of the synthesized copolymers were recorded at 25 °C on a 700 MHz Bruker DPX spectrometer. Chemical shifts (δ) are reported in ppm with the residual nondeuterated solvent signal as internal standard (chloroform at 7.26 and 77.16 ppm and dimethylsulfoxide at 2.51 and 39.52 ppm for ^1^H and ^13^C-NMR spectroscopy, respectively). The data were reported as follows: s = singlet, m = multiplet or unresolved, bs = broad singlet. Gel permeation chromatography (GPC) was performed in a chromatographic system (Waters Division Millipore) equipped with a Waters model 410 refractive-index detector using *N,N*-dimethylformamide (Scharlau, 99.9%) containing 0.1% of LiBr (Aldrich, 99.99%) as the eluent at a flow rate of 1 mL/min at 50 °C. Poly(methyl methacrylate) standards (Polymer Laboratories Ltd., Church Stretton, UK) were used to calibrate the columns. The zeta potential values of copolymers were analyzed using the Zetasizer Nano series ZS (Malvern Instrument Ltd.) at 25 °C from aqueous solutions at a concentration of 1 mg/mL.

### 2.3. Synthesis of Statistical Copolymers, P(PEGMA–*co*–MTZ)

**Scheme 1:**
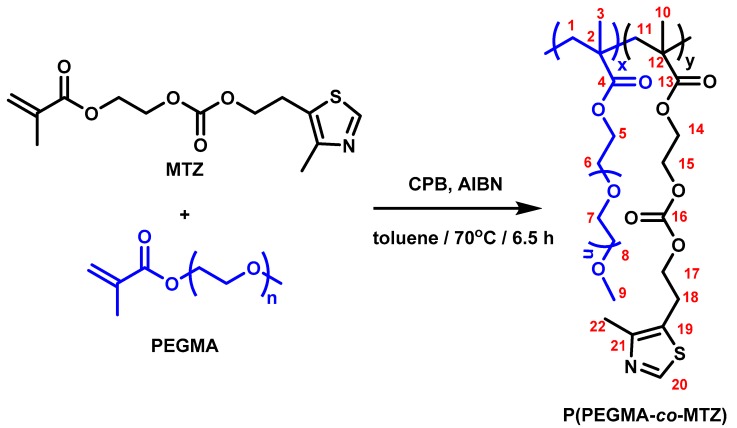
Reversible addition-fragmentation chain transfer (RAFT) copolymerization reaction of 2-(((2-(4-methylthiazol-5-yl)ethoxy)carbonyl)oxy)ethyl methacrylate monomer (MTZ) and poly(ethylene glycol) methyl ether methacrylate (PEGMA): preparation of P(PEGMA–*co*–MTZ) copolymers. CPB, 2-cyano-2-propyl benzodithioate; AIBN, 2,2’-Azobisisobutyronitrile.

A series of P(PEGMA–*co*–MTZ)s copolymers were synthesized via reversible addition-fragmentation chain transfer (RAFT) polymerization of PEGMA and MTZ monomers using different feed ratios (monomer molar ratios PEGMA/MTZ: 75/25, 50/50, and 25/75), as shown in Scheme 1. The corresponding homopolymers, PPEGMA (PEGMA/MTZ: 100/0) and PMTZ (PEGMA/MTZ: 0/100), were also synthesized for comparative purposes. Briefly, in a glass tube, both comonomers were introduced in a total concentration of 2.3 M in anhydrous toluene, varying the PEGMA/MTZ ratio to synthesize the copolymers of the P(PEGMA–*co*–MTZ) series. The chain transfer agent, CPB, and the initiator, AIBN, were subsequently added in a concentration adjusted to the molar ratio [Monomer]/[CPB]/[AIBN] = 100/2/1. The mixture was deoxygenated by purging argon for 30 min and then stirred at 70 °C for 6.5 h. Then, the resulting polymer was isolated by precipitation in pentane and dried under vacuum. Monomer conversions were calculated gravimetrically based on the monomer compositions in the feed and the molar compositions in the copolymers were analyzed by NMR. All the copolymers present almost similar chemical shifts and different relative intensities depending on the monomer molar ratio.

As an example, copolymer P(PEGMA–*co*–MTZ), PEGMA/MTZ 50/50 = ^**1**^**H NMR** (700 MHz, DMSO-d6) δ (ppm): 8.81 (s, 1H, H-20), 4.23 (bs, 4H, H-15; H-17), 4.12 (bs, 2H, H-14), 4.01 (bs, 2H, H-5), 3.58 (bs, 2H, H-6), 3.50 (bs, 10H, H-7), 3.42 (bs, 2H,H-8), 3.23 (s, 3H, H-9), 3.12 (s, 2H, H-18), 2.32 (s, 3H, H-22), 2.05–1.49 (m, 4H, H-1; H-11), 1.49–0.59 (m, 6H, H-3; H-10). **^13^C NMR** (176 MHz, DMSO-d6) δ (ppm): 177.16; 176.35 (C-4; C-13), 154.68 (C-16), 151.19 (C-20), 150.04 (C-21), 126.76 (C-19), 71.74 (C-8), 70.08; 70.19; 70.26; 70.32 (C-7), 68.25 (C-6), 67.88 (C-17), 65.55 (C-15), 64.30 (C-5), 62.86 (C-14), 58.49 (C-9), 54.11 (C-1; C-11), 44.90–44.53 (C-2; C-12), 25.48 (C-18), 18.49–16.62 (C-32; C-10), 15.00 (C-22).

### 2.4. Synthesis of Cationic P(PEGMA–co–MTZ-RI) Copolymers

**Scheme 2:**
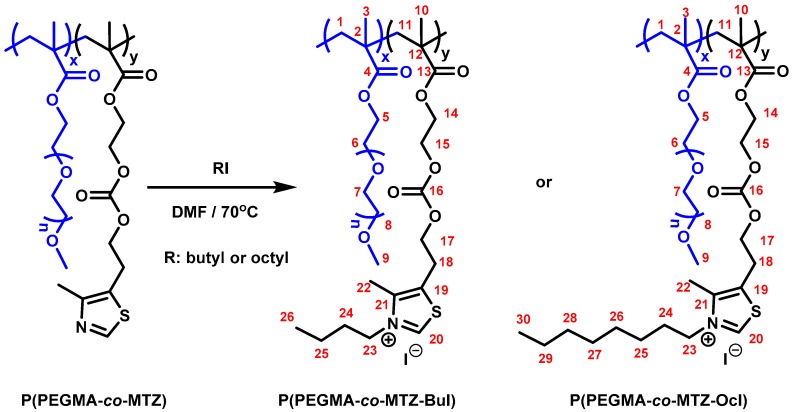
Quaternization reaction to prepare P(PEGMA–*co*–MTZ-BuI) and P(PEGMA–*co*–MTZ-OcI) cationic copolymers. DMF, *N,N*-dimethylformamide.

Quaternization reactions of the P(PEGMA–*co*–MTZ-RI) copolymers were carried out in glass tubes containing magnetic stirring bars. The copolymers were dissolved in anhydrous DMF and a large excess of alkyl iodide (molar ratio thiazole group/alkyl iodide 1:4), either 1-iodooctane or 1-iodobutane, was added (see Scheme 2). Mixtures were deoxygenated by purging argon for 15 min and heated at 70 °C. The degree of quaternization was followed from aliquots at different periods by NMR. After complete modification was achieved in each case, the resulting cationic copolymers were isolated by precipitation in hexane. A further purification step was carried out by newly solving the product in DMF, dialysis against distilled water, and finally freeze-drying under vacuum.

As happened with the unquaternized copolymer, the signals for each series appear at the same positions, but with different intensities depending on the composition. Therefore, as an example, the derivative of PEGMA/MTZ 50/50 is described.

Copolymer P(PEGMA–*co*–MTZ-BuI): PEGMA/MTZ 50/50 = **^1^H NMR** (700 MHz, DMSO-d6) δ (ppm): 10.1 (bs, 1H, H-20); 4.45 (bs, 2H, H-23); 4.28 (bs, 4H, H-15, H-17); 4.14 (bs, 2H, H-14); 4.00 (bs, 2H, H-5); 3.59 (bs, 2H, H-6); 3.49 (bs, 10H, H-7); 3.43 (bs, 2H, H-8); 3.31 (bs, 2H, H-18); 3.24 (s, 3H, H-9); 2.45 (bs, 3H, H-22); 2.10–1.45 (m, 4H, H-1, H-11); 1.45–1.15 (m, 4H, H-24, H-25); 1.15–0.59 (m, 6H, H-3, H-10. H-26). **^13^C NMR** (176 MHz, DMSO-d6) δ (ppm): 177.34, 173.51 (C-4, C-13); 157.47, 157.01 (C-16); 154.48 (C-20); 143.94, 143.37 (C-21); 133.88, 133.14 (C-19); 71.74 (C-8); 70.26, 70.17, 70.08 (C-7); 68.27 (C-6); 67.03 (C-17); 66.67 (C-15); 64.35 (C-5); 62.98 (C-14); 58.52 (C-9); 54.67–53.08 (C-1, C-11, C-23); 44.91, 44.53 (C-2, C-12); 31.62 (C-24); 26.02 (C-18); 22.54 (C-25); 18.40, 16.57 (C-3, C-10); 14.41 (C-26); 11.61 (C-22).

Copolymer P(PEGMA–*co*–MTZ-OcI): PEGMA/MTZ 50/50 = **^1^H NMR** (700 MHz, DMSO-d6) δ (ppm): 10.1(bs, 1H, H-20); 4.45 (bs, 2H, H-23); 4.29 (bs, 4H, H-15; H-17); 4.13 (bs, 2H, 14); 4.00 (bs, 2H, H-5); 3.58 (bs, 2H, H-6); 3.51 (s,10H, H-7); 3.43 (bs, 2H, H-8); 3.31 (s, 2H, H-18); 3.23 (s, 3H, H-9); 2.45 (bs, 3H, H-22); 2.12–1.42 (m, 4H, H-1; H-11); 1.40–1.15 (m, 12H, H-24, H-25, H-26, H-27, H-28, H-29); 1.08–0.63 (m, 6H, H-3; H-10); 0.85 (s, 3H, H-30). **^13^C NMR** (176 MHz, DMSO-d6) δ (ppm): 177.28, 176.28 (C-4, C-13); 157.44, 156.98 (C-16); 154.68, 154.47 (C-20); 143.93, 143.35 (C-21); 133.86, 133.13 (C-19); 71.74 (C-8); 70.25, 70.17, 70.08 (C-7); 68.25 (C-6); 67.03 (C-17); 65.68 (C-15); 64.33 (C-5); 62.97 (C-14); 58.50 (C-9); 54.23–52.16 (C-1; C-11); 53.46 (C-23); 44.91, 44.53 (C-2, C-12); 31.61, 29.30, 29.04, 28.96, 28.82 (C-24, C-25, C-26, C-27, C-28); 26.01 (C-18); 22.53 (C-29); 18.48, 16.59 (C-3, C-10); 14.39 (C-30); 11.58 (C-22).

### 2.5. Determination of the Antimicrobial Activity of the Copolymers 

Minimum inhibitory concentrations (MICs) of the copolymers against *S. aureus*, *S. epidermidis*, *E. coli*, *P. aeruginosa*, and *C. parapsilosis* were determined by following the Clinical Laboratory Standards Institute (CLSI) microbroth dilution reference method [24]. Microorganisms were cultured on 5% sheep blood Columbia agar for 24 or 48 h (bacteria or fungi, respectively) at 37 °C. Subsequently, microorganism suspensions of ∼2 × 10^8^ colony-forming units (CFU)/mL were prepared in sterile 0.9% saline solution. The inoculum was then diluted to achieve ~2 × 10^6^ CFU/mL in Mueller–Hinton broth. Each polymer was dissolved in a mixture of Mueller–Hinton broth and a minimum amount of DMSO (less than 5%, *v*/*v*, non-toxic for bacteria) at a concentration of 10 mg/mL. Subsequently, 50 μL of broth was pipetted into all the wells of a sterile 96-well microplate, except in the first column. Polymer solution (100 μL, 10 mg/mL) was added into the first column and was twofold serially diluted across the plate. The wells of the broth microdilution plates were inoculated with 50 μL of each test microorganism suspensions for a total volume of 100 μL to yield the standard density of ~5 × 10^5^ CFU/mL. Control wells containing culture controls without polymer were undertaken. The microplates were then incubated at 37 °C for 24 h for bacteria and 48 h for yeast, respectively. Afterward, MIC values were determined as the lowest polymer concentration at which visible growth of the microorganism was inhibited. All assays were tested in triplicate.

### 2.6. Hemolysis Assay

The hemotoxicity of synthesized polymers was studied on fresh human blood obtained from healthy donors [20]. Briefly, a blood sample was extracted and collected in blood collecting tubes containing ethylenediamine tetraacetic acid (EDTA) as anticoagulant (Vacuette^®^ K3E EDTA K3, Greiner Bio-One International). Then, the tubes were centrifuged immediately at 3500 rpm for 20 min. Supernatant (plasma) and the buffy coat at the middle (white blood cells) were removed, whereas red blood cells (RBC) at the bottom were isolated and washed with fresh sterile PBS and centrifuged three times. Subsequently, a 5% *v*/*v* erythrocyte suspension was prepared in PBS and used as working suspension. Each polymer was dissolved in a mixture of PBS and a minimum amount of DMSO (less than 5 vol %, demonstrated to be nontoxic at the experimental conditions used in this study) at a concentration of 20 mg/mL. These polymer solutions (100 μL) were placed in the first column of 96-well round-bottom microplates, and in the rest of the wells, 50 μL of PBS was added. Then, twofold sequential dilutions of the polymer solutions were carried out to obtain series concentrations. Then, 150 μL of RBC suspension was added to each well. Triton X-114 (1% *v*/*v* solution in PBS) and PBS were used as positive control for 100% hemolysis and negative control for 0% hemolysis, respectively. The microplate was incubated for 1 h at 37 °C, and centrifuged at 1000 rpm during 10 min. The supernatant was transferred to empty 96-well microplates to measure the absorbance at 550 nm using a microplate reader (VirClia^®^ Chemiluminescence). Absolute achromatic supernatant solution means no hemolysis, while red solution implies hemolysis. The percentage of hemolyzed erythrocytes was calculated according to the following equation:

$$\mathrm{Hemolysis}\;\%=\frac{A_{\mathrm{sample}}-A_{\mathrm{negative control}}}{A_{\mathrm{positive control}}-A_{\mathrm{negative control}}}\times 100$$

All experiments were done in triplicate, and the data were expressed as mean ± SD (*n* = 3).

## 3. Results and Discussion

**Figure 1:**
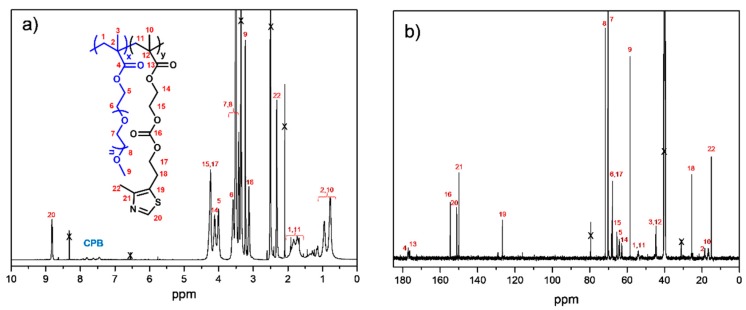
(**a**) ^1^H and (**b**) ^13^C nuclear magnetic resonance (NMR) spectra of the copolymer with a PEGMA/MTZ ratio of 50/50.

A series of statistical copolymers of PEGMA and MTZ, P(PEGMA–*co*–MTZ), were synthesized via control RAFT polymerization with different monomer molar ratios PEGMA/MTZ: 100/0, 75/25, 50/50, 25/75, and 0/100 in the feed. RAFT polymerization was employed to ensure control over the molecular weight and polydispersity. All the reactions were carried out during 6.5 h at 70 °C to assure high conversion. As an example, Figure 1 shows the ^1^H and ^13^C NMR spectra of the copolymer with a PEGMA/MTZ ratio of 50/50, in which both components can be clearly identified. ^1^H NMR spectroscopy was employed to measure the composition of the copolymers by the ratio of the integrals of the resonance signals related to each monomeric unit: two methylene protons of MTZ at 3.1 ppm (H18, Scheme 1) and the combined resonances of the methylene protons of MTZ and PEGMA in the range of 4.5–4.0 ppm (H5, H14, H15, H17, see Scheme 1, after subtracting the contribution from methylene protons from MTZ units). The presence of the CPB end group in the polymers was confirmed by ^1^H NMR spectroscopy. Besides, ^1^H NMR was used to calculate the number average molecular weights (*M*_n_) of copolymers by comparing the integration values of chain end phenyl protons of CPB agent at 7.5–7.9 ppm to the characteristic protons from PEGMA (H9 at 3.25 ppm) and MTZ (H18 at 3.1 ppm) units. The molecular characteristics of the copolymers are given in Table 1. The final molar compositions of the resulting copolymers are close to feed molar composition owing to high conversion, although the percentage of MTZ is slightly smaller in the final copolymers.

**Figure 2:**
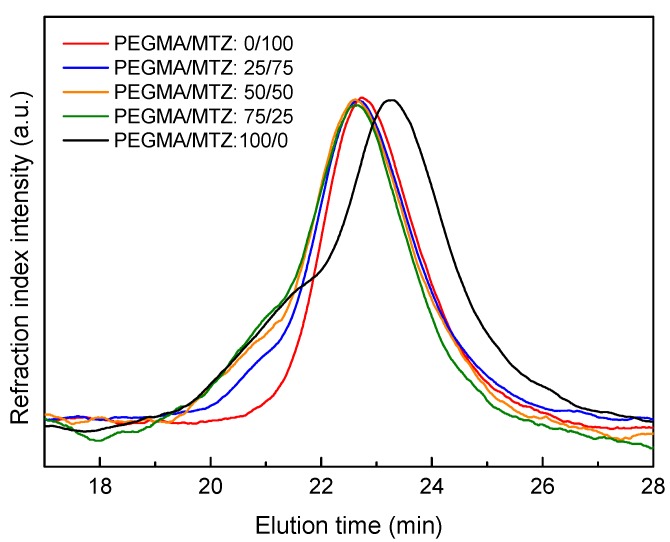
Gel permeation chromatography (GPC) curves of the P(PEGMA–*co*–MTZ) copolymers synthesized via reversible addition-fragmentation chain transfer (RAFT) polymerization with different monomer molar ratios PEGMA/MTZ: 100/0, 75/25, 50/50, 25/75, and 0/100 in the feed.

The *M*_n_ values of the synthesized polymers were also determined by GPC. The data, summarized in Table 1, show that molecular weights determined by GPC are slightly lower than the values obtained by NMR, probably owing to the mismatch between the PMMA standards used for the GPC calibration and the prepared polymers. All the polymers present low polydispersity indexes, indicating that the polymerization proceeded in a control manner. However, polydispersity increases as the content of PEGMA units augments in the copolymer. The GPC curves displayed in Figure 2 show high molecular weight shoulders for polymers containing PEGMA units, which consequently provoke the broadening of the curves. This is probably associated to chain transfer processes, which can lead to branched structures [25].

**Figure 3:**
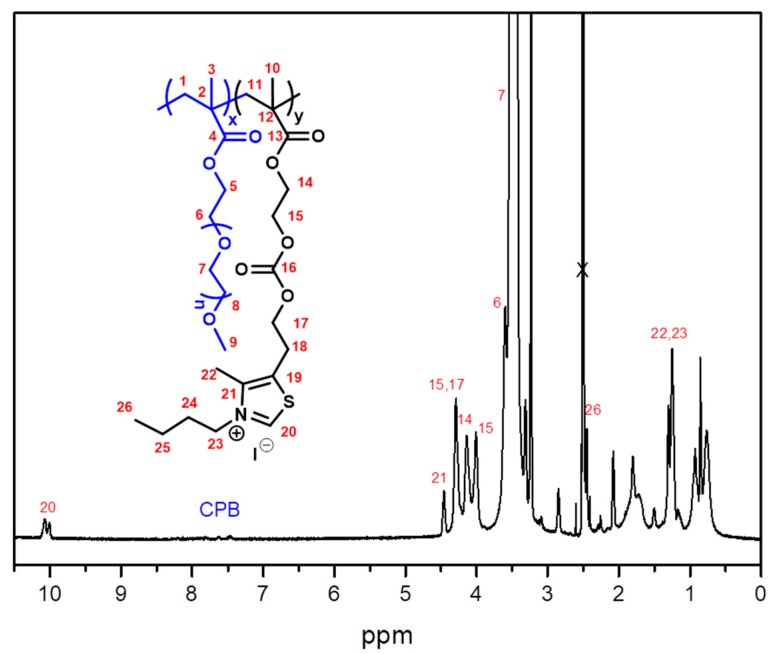
^1^H NMR spectrum of the copolymer quaternized with 1-iodobutane, P(PEGMA–*co*–MTZ-BuI), for a PEGMA/MTZ ratio of 50/50.

From these synthesized copolymers containing thiazole moieties in the MTZ units, cationic copolymers were subsequently prepared by quaternization reaction using alkylating agents. For this purpose, two different hydrophobic alkylating agents were employed, 1-iodooctane (C_8_) and 1-iodobutane (C_4_), in order to provide cationic charges and increase the hydrophobicity, as the hydrophobic/hydrophilic balance of these polymers is usually a key factor to achieve pronounced activity against bacteria and low toxicity to mammalian cells [12,18,26]. The reaction was carried out at 70 °C in sealed glass tubes during long periods of time to achieve complete quaternization of nitrogen from the thiazole ring. In the case of 1-iodobutane, a reaction time of 14 days was necessary, whereas for 1-iodooctane, longer periods (21 days) were required owing to its larger size. Figure 3 displays the ^1^H NMR spectrum of the copolymer quaternized with 1-iodobutane, P(PEGMA–*co*–MTZ-BuI), for a PEGMA/MTZ ratio of 50/50. A new peak at around 10.1 ppm was clearly observed, which was attributed to the proton of thiazolium ring (H-20, Scheme 2), while the peak at ca. 8.8 ppm, assigned to the proton of unquaternized thiazole group (H-20, Scheme 1), totally disappears from the spectrum. Similar spectra are obtained for all quaternized copolymers.

The obtained cationic copolymers were studied in aqueous media by zeta potential measurements. The prepared aqueous solutions in distilled water (1 mg/mL) were found to be stable for long periods. The data reported in Table 2 show that, in effect, all of the samples have a positive zeta potential value. This value becomes more positive as the content of MTZ units augments in the copolymers, because more thiazolium groups are presented. Moreover, it is appreciated that the samples quaternized with 1-iodooctane exhibit slightly higher zeta potential values, meaning that the positive charges are more exposed, probably as a consequence of the aggregation behavior of these samples with a longer hydrophobic chain.

The antimicrobial activity of the alkylated copolymers was evaluated using the broth dilution method [24] against Gram-positive bacteria (*S. aureus*, *S. epidermidis*), Gram-negative (*E. coli*, *P. aeruginosa*) bacteria, and yeast (*C. parapsilosis*), and reported as minimum inhibitory concentrations (MICs) (see Table 3). Remarkably, the PMTZ-BuI homopolymer (PEGMA/MTZ-BuI: 0/100) exhibits moderate antimicrobial activity, with MIC values much higher than those reported in a previous publication for a polymer with the same structure (MIC values between 4 and 8 µg/mL) [12]. This effect may be related with the molecular weight, as the homopolymer PMTZ-BuI of the current work has an *M*_n_ of 13,800 g/mol, while the previously reported polymer presented an *M*_n_ of 78,300 g/mol. It is well known that the molecular weight of cationic amphiphilic polymers can affect the antimicrobial activity [1,27,28,29], and in this case, it seems that low molecular weights demerit the antimicrobial performance against all the tested microorganisms. However, polymers with high molecular weights are typically more toxic because they could induce gen transfection, hemolysis, and hemagglutination, and are compounds that are difficult to eliminate by the kidney [30]. Regarding the influence of the hydrophobicity, the alkylation reaction with iodides of different lengths (butyl or octyl) allows us to vary hydrophobicity, keeping constant the hydrophilic and cationic groups of the copolymer. As seen in Table 3, the zeta potential of the polymers analyzed in the current work is, in general, higher for the samples alkylated with octyl iodide. Typically, as the hydrophobicity of the polymers augments, the antimicrobial activity tends to improve [31]; although, if the hydrophobicity increases so much that the solubility is compromised, the polymers aggregate and become unavailable to interact with the bacterial membrane. In addition, with increased hydrophobicity, the polymers become toxic not only to bacteria, but also to mammalian cells. Thus, the balance between cationic hydrophilic and hydrophobic groups is a crucial factor in the design of antimicrobial polymers. The incorporation of non-ionic hydrophilic units such as PEGMA also significantly affects the antimicrobial efficacy of the polymers. Clearly, the activity is markedly reduced as the content of PEGMA increases in the copolymers. An increment in the molar fraction of PEGMA decreases the number of cationic and hydrophobic side chains, and leads to lower electrostatic and lipophilic interactions between the microbial cell surfaces and polymers [17,19,32]. This effect is dramatic in the case of butylated copolymers, in which the hydrophobic/hydrophilic balance is highly affected and, consequently, the antimicrobial efficiency. In contrast, the series of copolymers with a long alkyl chain, PEGMA/MTZ-OcI, maintains relatively good levels of activity, with MIC values of 312 µg/mL or lower against Gram-positive bacteria and yeast, even with a molar percentage of PEGMA of up to 75%.

From the results, Gram-negative bacteria seem to present higher sensitivity to PEGMA incorporation in these series of copolymers. The decrease of activity owing to PEGMA addition is more evident against Gram-negative bacteria than against Gram-positive. Similar behaviors have been found in the literature [18], although other studies concluded in lowering activity of PEGylated polymers against Gram-positive bacteria such as *S. aureus* [17].

Then, a hemolysis test was performed to evaluate the toxicity against human eukaryotic cells of the prepared cationic copolymers. This is often studied with RBCs by determining the HC_50_ value, the value at which 50% of RBCs are lysed upon exposure to the polymer within an incubation period of one hour [31,33]. Besides, the standard of non-hematotoxicity usually refers to a hemolysis percentage below 5% (HC_5_). Both parameters are displayed in Table 4.

**Figure 4:**
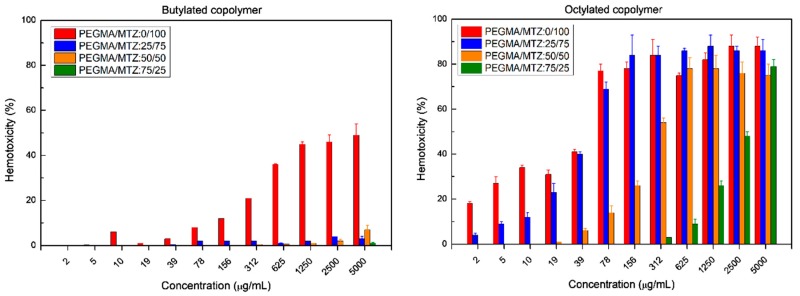
Hemotoxicity behavior of copolymers against red blood cells.

As represented in Figure 4 and collected in Table 4, the butylated copolymers exhibit a lower hemolysis percentage than the copolymers quaternized with octyl iodide, and do not exhibit the HC_50_ characteristic at any concentration below 5000 µg/mL for all polymeric compositions. Then, the values are larger than this highest concentration of compound tested at 5000 µg/mL. In contrast, octylated copolymers present HC_50_ at a concentration smaller than 39 µg/mL. Therefore, although the copolymers containing a longer hydrophobic chain, C_8_, have better antimicrobial activity, they are much more hemotoxic than their corresponding copolymers quaternized with butyl iodide, as hydrophobic groups are known to be responsible for inducing hemolysis capacity [13,18]. The cell surface of eukaryotic cells is mainly composed of zwitterionic lipid head groups and cholesterol and lacks a net negative charge; thus, the hemolytic activity of the copolymers principally arises from the hydrophobic interactions between the hydrophobic groups in polymers and the lipid bilayer of RBCs [15,34].

The incorporation of PEGMA units in the copolymer structure markedly reduces the hemolytic activity. For both series of copolymers, as the content of PEGMA augments in the copolymer, the hemolysis percentage decreases. P(PEGMA–*co*–MTZ-BuI) copolymer with PEGMA/MTZ-BuI ratio = 50/50 at a concentration as high as 5000 µg/mL exhibits a very low hemolysis percentage, less than 5%, which is similar to the negative control, and considered no-hemotoxic. Only 25% of PEGMA units contribute to the remarkable hemocompatibility obtained in the copolymers at concentrations below 2500 µg/mL.

Table 4 also summaries selectivity values of the copolymers that refer to the preferential activity of the polymer against pathogens over mammalian cells, and are typically expressed by taking the ratio of the HC_50_ versus the MIC values. In the series of butylated copolymers, the incorporation of PEGMA, in general, reduces the selectivity owing to the drastic decrease of the antimicrobial activity. Although the inclusion of PEGMA units at the molar percentages tested does not improve the selectivity, it clearly enhances the hemocompatibility, fulfilling the standard of non-hematotoxicity HC_5_ at a concentration higher than 5000 µg/mL.

On the other hand, for the octylated copolymers, the addition of a high content of PEGMA units, 75% mol, improves the selectivity values for Gram-positive and yeast strains, mainly because it significantly reduces hemotoxicity.

## 4. Conclusions

In conclusion, a series of cationic copolymers with amphiphilic monomeric units were synthesized and their antimicrobial activity against Gram-negative, Gram positive bacteria, and yeast was tested. The hydrophobic/hydrophilic balance was varied through quaternization reaction of thiazole moiety with either butyl or octyl chains. Copolymers with higher hydrophobicity owing to long octyl groups manifested higher antimicrobial activity because the hydrophobic interactions with cell surface increased, and thus the cell membrane permeability of copolymers. However, the hemotoxicity was also highly affected in copolymers quaternized with octyl alkylating agents. Further incorporation of hydrophilic PEGMA units to the polymers led to a substantial reduction of hemolytic activity in both families of polymers, but the antimicrobial activity was also seriously reduced. Whereas the addition of PEGMA improved selectivity in octylated copolymers, the selectivity values in butylated copolymers worsened. Tuning polymer amphiphilicity through the nature of the alkyl side chains and the incorporation of hydrophilic and non-hemolytic PEGMA dramatically changes the biological activity of the copolymer, and emphasizes the role of optimum amphiphilicity in the development of active antibacterial and nonhemolytic polymers.

## Figures and Tables

**Table 1:** Molecular parameters of the synthesized polymers: copolymer composition poly(ethylene glycol) methyl ether methacrylate (PEGMA)/2-(((2-(4-methylthiazol-5-yl)ethoxy)carbonyl)oxy)ethyl methacrylate monomer (MTZ), conversion, number average molecular weight (*M*_n_) determined by nuclear magnetic resonance (NMR) and gel permeation chromatography (GPC), and polydispersity index (PDI) determined by GPC.

Feed PEGMA/MTZ	Copolymer PEGMA/MTZ	Conversion (%)	*M*_n_^NMR^ (g/mol)	*M*_n_^GPC^ (g/mol)	PDI
100/0	100/0	99	13,500	10,500	1.23
75/25	79/21	99	20,400	13,000	1.14
50/50	58/42	98	19,900	12,300	1.14
25/75	33/67	98	17,300	11,700	1.12
0/100	0/100	96	13,800	11,200	1.09

**Table 2:** Zeta potential values of the cationic copolymers P(PEGMA–*co*–MTZ-BuI) and P(PEGMA–*co*–MTZ-OcI).

Copolymer PEGMA/MTZ-BuI	Zeta Potential (mV)	Copolymer PEGMA/MTZ-OcI	Zeta Potential (mV)
75/25	14	75/25	12
50/50	25	50/50	26
25/75	42	25/75	50
0/100	50	0/100	59

**Table 3:** Antimicrobial activities of the cationic copolymers P(PEGMA–*co*–MTZ-BuI) and P(PEGMA–*co*–MTZ-OcI). MIC, minimum inhibitory concentration.

Sample	MIC (µg/mL)				
	*S. aureus*	*S. epidermidis*	*P. aeruginosa*	*E. coli*	*C. parapsilosis*
PEGMA/MTZ-BuI 75/25	>5000	5000	5000	>5000	>5000
PEGMA/MTZ-BuI 50/50	5000	2500	5000	>5000	5000
PEGMA/MTZ-BuI 25/75	5000	312	5000	>5000	5000
PEGMA/MTZ-BuI 0/100	2500	156	625	156	2500
PEGMA/MTZ-OcI 75/25	312	312	2500	5000	312
PEGMA/MTZ-OcI 50/50	312	78	2500	5000	312
PEGMA/MTZ-OcI 25/75	156	39	39	78	312
PEGMA/MTZ-OcI 0/100	78	19	39	39	39

**Table 4:** Hemolytic activities (HC_50_) and selectivity values (HC_50_/MIC) obtained for the cationic copolymers P(PEGMA–*co*–MTZ-BuI) and P(PEGMA–*co*–MTZ-OcI). HC_5_, hemolysis percentage below 5%.

Sample	HC_5_ (µg/mL)	HC_50_ (µg/mL)	HC_50_/MIC				
			*S. aureus*	*S. epidermidis*	*P. aeruginosa*	*E. coli*	*C. parapsilosis*
PEGMA/MTZ-BuI 75/25	>5000	>5000	-	>1	>1	-	-
PEGMA/MTZ-BuI 50/50	>5000	>5000	>1	>2	>1	-	>1
PEGMA/MTZ-BuI 25/75	>5000	>5000	>1	>16	>1	-	>1
PEGMA/MTZ-BuI 0/100	39	>5000	>2	>32	>8	>32	>2
PEGMA/MTZ-OcI 75/25	312	2500	8	8	1	0.5	8
PEGMA/MTZ-OcI 50/50	19	312	1	4	<0.12	<0.06	1
PEGMA/MTZ-OcI 25/75	2.4	39	<0.25	1	1	0.5	<0.12
PEGMA/MTZ-OcI 0/100	<2.4	39	<0.5	2	1	1	1
